# Mol­ecular and crystal structure of *catena*-poly[1-benzyl-[1,2,4]triazolo[1,5-*c*]quinazolin-1-ium-2,5-bis­(thiol­ate) [[aqua­sodium]-di-μ-aqua]]

**DOI:** 10.1107/S2056989026005487

**Published:** 2026-05-29

**Authors:** Svitlana V. Shishkina, Maryna S. Kovalenko, Oleksandr G. Drushlyak, Mariia O. Shyshkina, Christian Dank, Sergiy M. Kovalenko

**Affiliations:** a Institute of Organic Chemistry, NAS of Ukraine, Akademik Kukhar Street 5, Kyiv 02094, Ukraine; b Institute of Organic Chemistry, University of Vienna, Wahringer Strasse 38, 1090 Vienna, Austria; c V. N. Karazin Kharkiv National University, Svobody sq. 4, Kharkiv 61077, Ukraine; d Institute of Low Temperature and Structure Research Polish Academy of Sciences, Okolna 2 50- 422 Wroclaw Poland; e Faculty of Chemistry Wroclaw University of Science and Technology, Wybrzeze Wyspianskiego 27 50-370 Wroclaw Poland; f Department of Pharmaceutical Sciences, University of Vienna, Josef-Holaubek-Platz 2 (UZA II), 1090 Vienna, Austria; Vienna University of Technology, Austria

**Keywords:** crystal structure, heterocycles, thiole, ylide, mesoionic structures, sodium salt

## Abstract

In the crystal structure of the title salt, {[Na(H_2_O)_3_](C_16_H_11_N_4_S_2_)}_*n*_, the 1-benzyl-[1,2,4]triazolo[1,5-*c*]quinazolin-1-ium-2,5-bis­(thiol­ate) anion is not included in the coordination sphere of the sodium cation. The latter is coordinated by five water mol­ecules, two pairs of which form bridges with two neighbouring sodium cations, whilst the fifth water mol­ecule additionally coordinates monodentately. The anion structure can be described as a superposition of two zwitterionic structures with two negative charges located on the sulfur atoms and one positive charge either on the nitro­gen nodal atom or on the nitro­gen atom bonded to the benzyl group. In the crystal structure, the organic mol­ecules are linked to the aqua ligands *via* inter­molecular O—H⋯(N,S) hydrogen bonds. The crystal packing can be characterized as layered, defining a three-layer unit parallel to (100) as the main motif. The central part of the three-layer unit consists of ^1^_∞_[Na(H_2_O)_1/1_(H_2_O)_4/2_] chains extending parallel to [010]. Two layers of anion mol­ecules sandwich the cation layer.

## Abstract 2

The crystal structure of the title compound shows a layered arrangement stacked along [100] where layers of ^1^_∞_[Na(H_2_O)_1/1_(H_2_O)_4/2_] chains are sandwiched by layers of mol­ecular anions.

## 1. Chemical context

Mesoionic heterocyclic compounds based on the 1,2,4-triazole core attract considerable attention due to their unusual electronic structure, a high degree of charge delocalization, and their ability to participate in various chemical transformations. The triazole fragment is widely used in medicinal chemistry, catalysis, and in the design of functional materials, as it is stable, easily modifiable, and provides favorable pharmacophoric properties (Couto Rodrigues *et al.*, 2025 ▸; Aggarwal & Sumran, 2020 ▸; El-Sebaey, 2020 ▸). A particularly important subclass of such systems is represented by mesoionic 1,2,4-triazolium-3-thiol­ates, in which the positively charged triazolium ring is conjugated with a thiol­ate entity. This structural arrangement results in a non-classical distribution of electron density and determines the characteristic reactivity, including alkyl­ation (Molina *et al.*, 1984 ▸; Wasfy, 2003 ▸), metal coordination (Shum *et al.*, 2025 ▸), or transformations into other heterocyclic systems (Reissig & Zimmer, 2014 ▸). In addition, these compounds often exhibit valuable physicochemical properties, such as high crystallinity and a pronounced dipole moment (Badami, 2006 ▸).

Condensed mesoionic derivatives, in which the triazole ring is annulated with a benzene ring or another aromatic moiety, remain insufficiently explored. Nevertheless, available data indicate that benzannulation enhances the stability of the mesoionic system and may broaden its reactivity profile. Several representatives of this class have already demonstrated anti­bacterial (Liu *et al.*, 2019 ▸), anti­thrombotic (Rehse *et al.*, 1994 ▸), anti­cancer (Brown *et al.*, 2018 ▸), and anti-inflammatory (Cardoso *et al.*, 2004 ▸) activities. Moreover, certain 1,3,4-thia­diazo­lium mesoionic compounds exhibit cytotoxicity toward melanoma cells, presumably due to their influence on cellular membranes (Senff-Ribeiro *et al.*, 2004 ▸; Cadena *et al.*, 2002 ▸). Particularly promising are iridium complexes derived from triazolo[1,5-*c*]quinazoline scaffolds, which display exceptionally high phototoxicity and are considered potential agents for photodynamic therapy (Shum *et al.*, 2025 ▸).

In previous studies, we developed an efficient one-step method for the synthesis of 1-substituted 5-thioxo-5,6-di­hydro-[1,2,4]triazolo[1,5-*c*]quinazolin-1-ium-2-thiol­ates based on the reaction of 2-iso­thio­cyanato­benzoates with thio­semicarbazides (Kovalenko *et al.*, 2020 ▸). In this context, it was revealed that 1-benzyl-[1,2,4]triazolo[1,5-*c*]quinazolin-1-ium-2,5-bis­(thiol­ate) crystallized as dimethyl formamide (DMF) or di­methyl­sulfoxide (DMSO) solvates. Their mol­ecular structures were compared to the structure of deprotonated 1-phenyl-[1,2,4]triazolo[1,5-*c*]quinazolin-1-ium-2,5-bis­(thiol­ate) coordinating to an iridium cation and compensating it charge in the intrinsic coordination sphere (Kovalenko *et al.*, 2026 ▸).
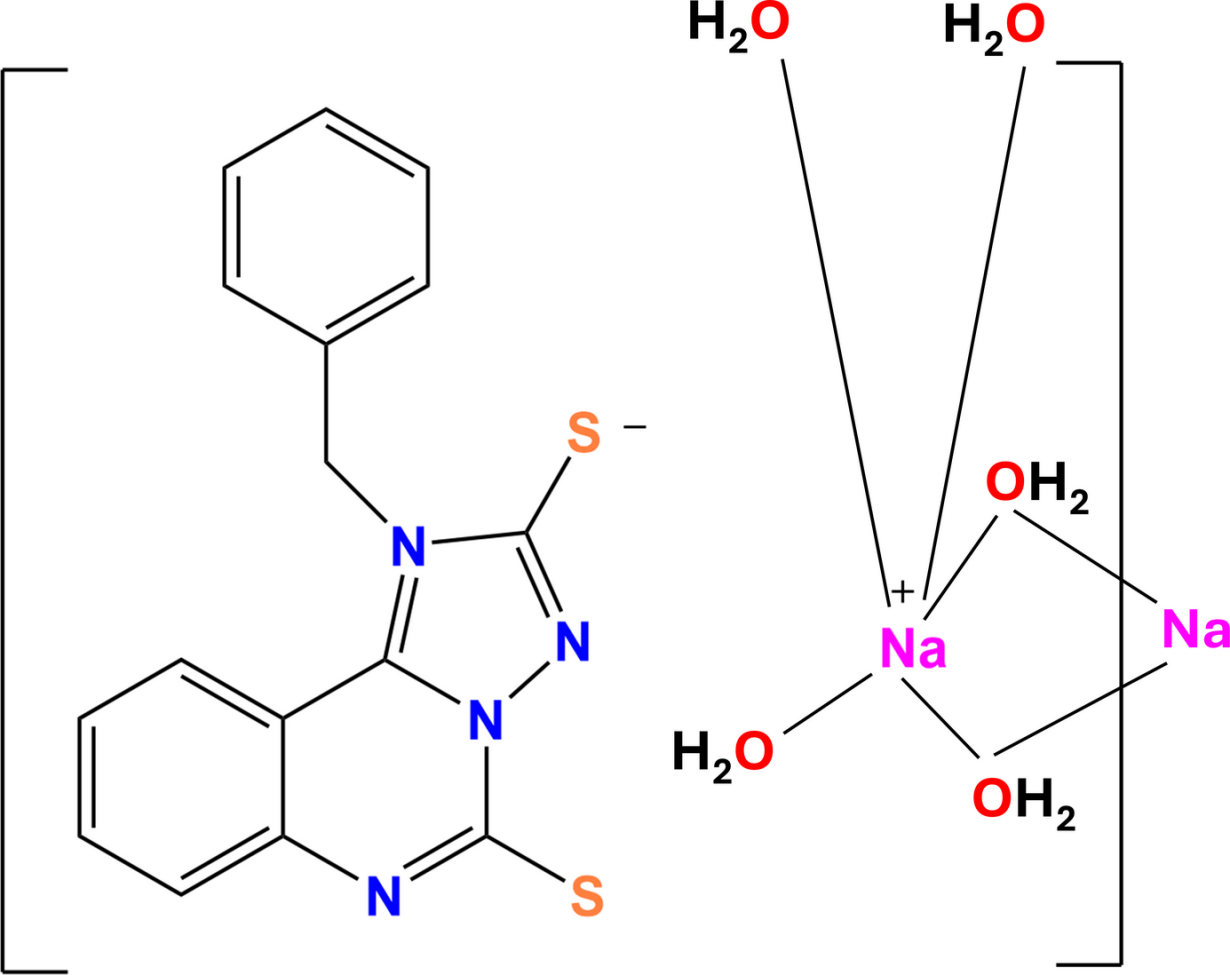


**Figure 1:**
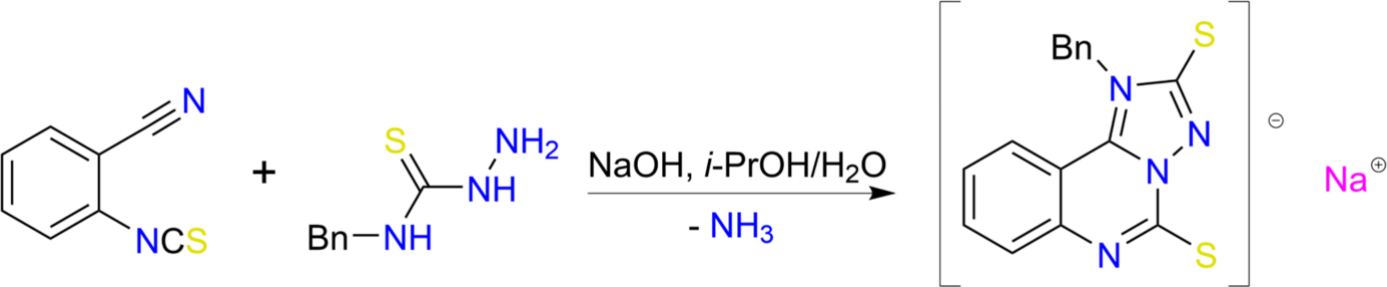
Synthesis scheme to obtain sodium 1-benzyl-[1,2,4]triazolo[1,5-*c*]quinazolin-1-ium-2,5-bis­(thiol­ate).

In the present work, we report the synthesis and single crystal X-ray diffraction study of the hydrated hybrid sodium salt of 1-benzyl-[1,2,4]triazolo[1,5-*c*]quinazolin-1-ium-2,5-bis­(thiol­ate) (**I**) where the organic anion compensates the positive charge of the cation outside its coordination sphere. The title compound was synthesized by the reaction of 2-iso­thio­cyanato­benzo­nitrile with *N*-benzyl­thio­semicarbazide in the presence of excess NaOH in a water–iso­propanol medium (Fig. 1 ▸). The sodium salt of (**I**) is a promising building block containing thiol­ate groups that can react with various alkyl­ating reagents, which provides convenient access to various new heterocyclic derivatives important for pharmaceuticals, veterinary medicine and agrochemistry.

## 2. Structural commentary

**Figure 2:**
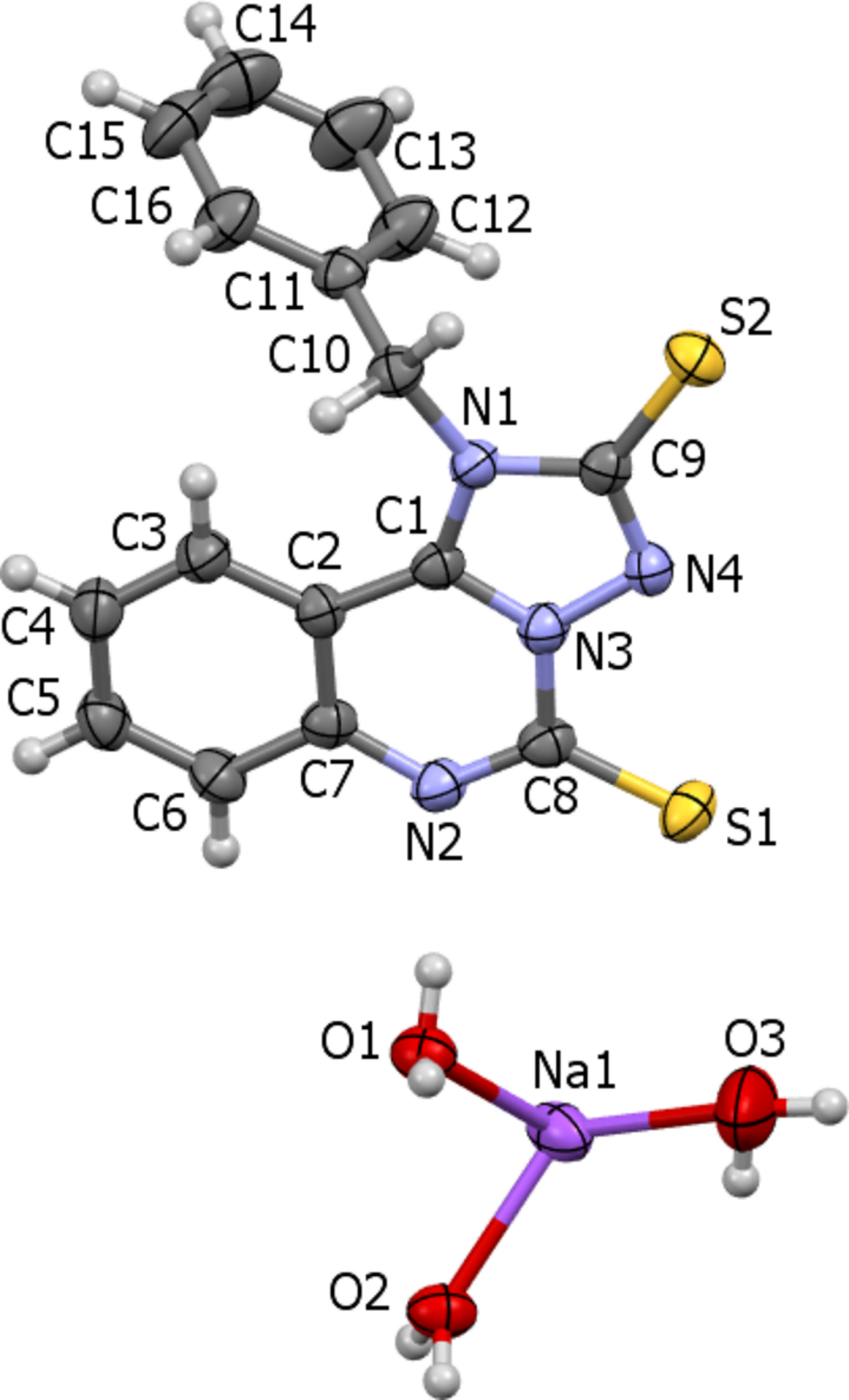
Mol­ecular structure of the sodium salt of (**I**), showing the asymmetric unit with atom labelling. Displacement ellipsoids are drawn at the 50% probability level.

**Figure 3:**
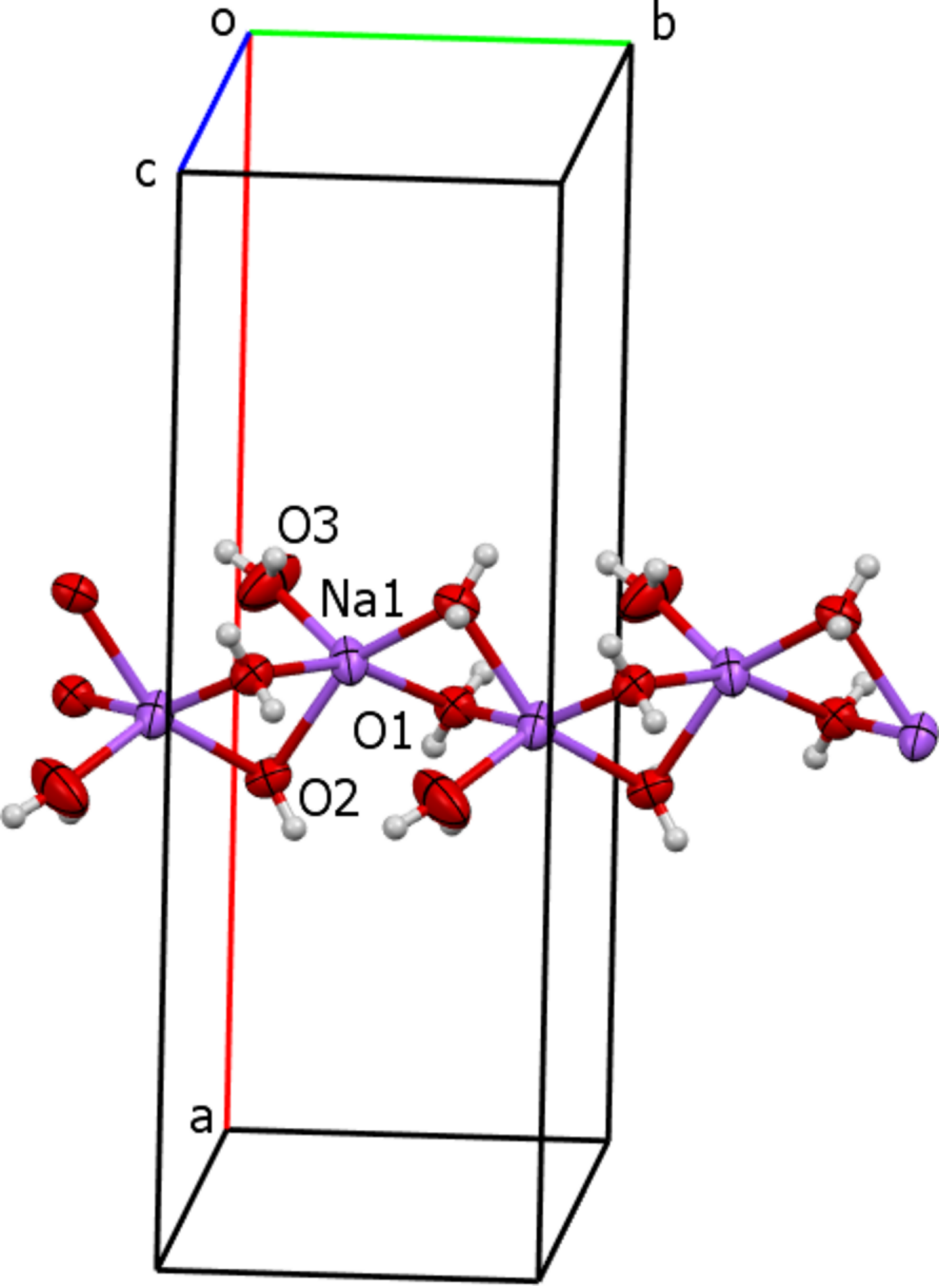
Sodium cations coordinated by water mol­ecules, resulting in a ^1^_∞_[Na(H_2_O)_1/1_(H_2_O)_4/2_] chain extending parallel to [010].

Crystallization of the sodium salt of compound (**I**) from aqueous DMF results in receiving of its trihydrate (Fig. 2 ▸). Two of the water mol­ecules bridge the sodium cations while the third water mol­ecule monodentately coordinates the sodium cation, leading to a polymeric chain^1^_∞_[Na(H_2_O)_1/1_(H_2_O)_4/2_] extending parallel to [010] (Fig. 3 ▸). The coordination sphere of the Na cation comprises five water mol­ecules and can be described as a square pyramid where the bridging oxygen atoms (two atoms O1 and two atoms O2) lie in a square base [root-mean-square (r.m.s.) deviation from the plane is 0.03 Å], while the O3 atom acts as an apical ligand (Fig. 3 ▸). The organic anion is not included in the sodium coordination sphere.

**Figure 4:**
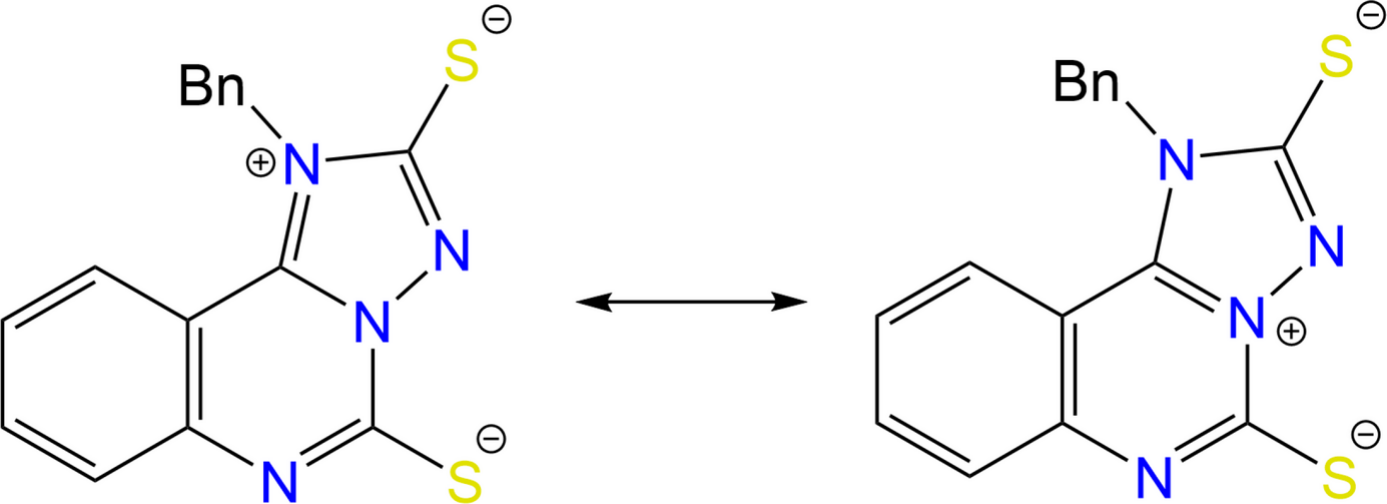
Zwitterionic resonance structures of the anion of (**I**).

The comparison of bond lengths in the anion of the sodium salt of (**I**) in the present structure with those in the previously studied neutral mol­ecules of (**I**) (Kovalenko *et al.*, 2026 ▸) and a similar anion coordinating to Ir^3+^ (Shum *et al.*, 2025 ▸) showed some differences in the electron density distribution within the heterocyclic fragment. In contrary to the anion of (**I**) in the complex with iridium, both C*sp*^2^=S bonds in the non-coordinating anion are elongated (Table 1 ▸) compared to the mean value of 1.671 Å (Bürgi & Dunitz, 1994 ▸). This allows to assume that the negative charges are located on the two sulfur atoms. One of these two negative charges is compensated by a positive charge in the anion. The C1—N1 and C1—N3 bonds are longer than the N4—C9 and N2—C8 double bonds, and shorter than the N1—C9 and N3—C8 bonds (Table 1 ▸). It can therefore be assumed that the C1—N1 and C1—N3 bonds are inter­mediate between a single C—N bond and a double C=N bond. Thus, the mol­ecular structure of anion of (**I**) can be described as a superposition of two resonance zwitterionic structures (Fig. 4 ▸), in which the positive charge is located either on the N1 atom or on the N3 atom.

The tricyclic fragment of the anion (**I**) is planar with an r.m.s. deviation of 0.015 Å. The phenyl group of the benzyl substituent is almost orthogonal to the C1–N1 endocyclic bond (the C1—N1—C10—C11 torsion angle is −80.1 (3)°) and is rotated around the C10–C11 bond in such a way that the dihedral angle between its aromatic plane and the tricyclic fragment is 85.85 (8)°.

## 3. Supra­molecular features

**Figure 5:**
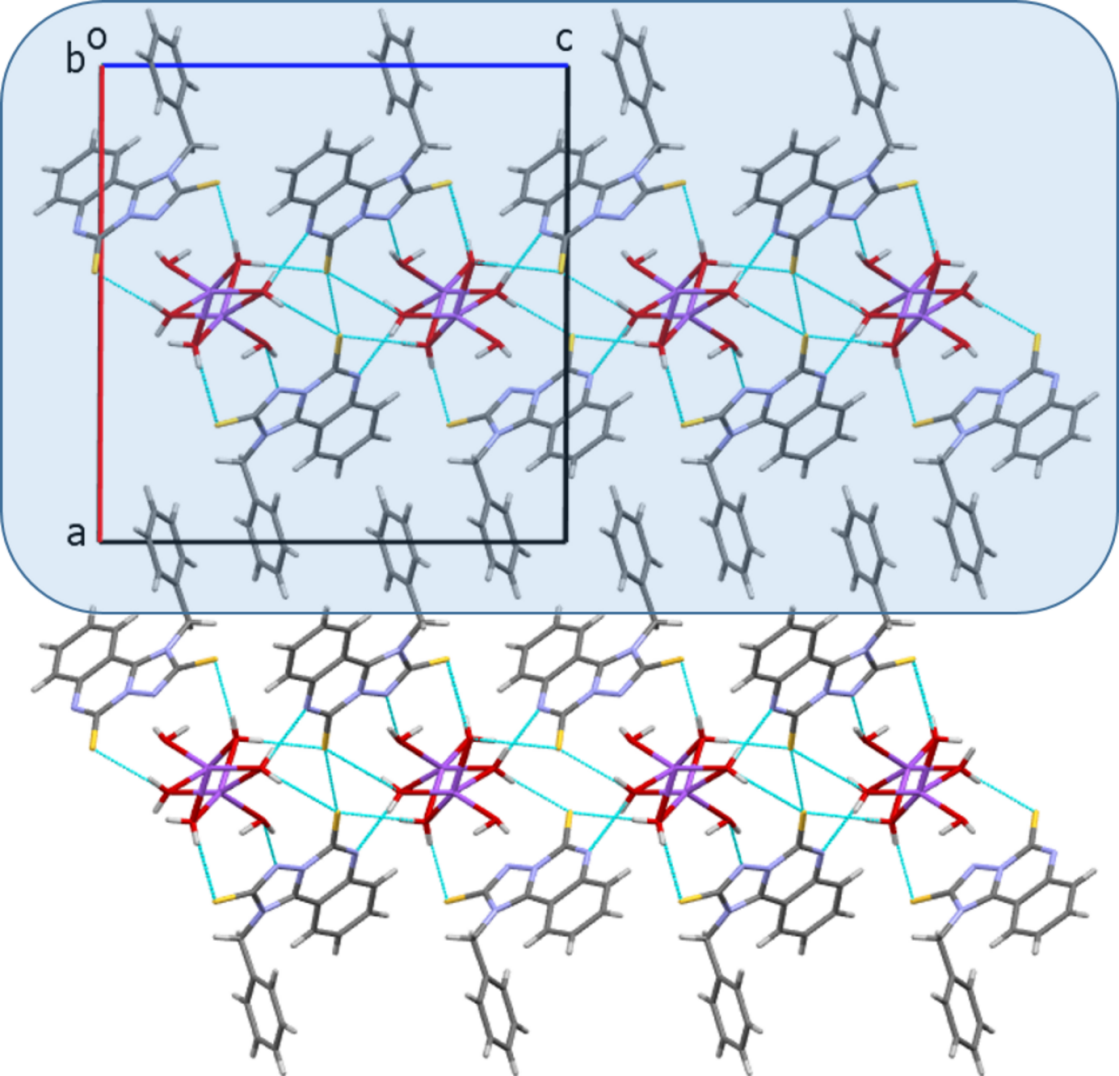
Crystal packing of the sodium salt of (**I**) in a projection along [010]. Inter­molecular hydrogen bonds are shown as blue dashed lines. A three-layer unit is highlighted.

The resonance structures of the anion (**I**) are additionally stabilized by inter­molecular hydrogen bonds between the aqua ligands as donors and N and S atoms of the anion as acceptors (Table 2 ▸). The ^1^_∞_[Na(H_2_O)_1/1_(H_2_O)_4/2_] chains form layers parallel to (100). There are layers of anions linked by hydrogen bonds to the water mol­ecules above and below the layer of cations (Fig. 5 ▸). Such three-layer units can be recognized as a main structural motif of the crystal packing. Additional stabilization results from weak C—H⋯S (Table 2 ▸) and π–π stacking inter­actions between the triazole ring and the benzene ring of the quinoline moiety of a neighbouring mol­ecule (symmetry code *x*, −1 + *y*, *z*), with a centroid-to-centroid distance of 3.9439 (16) Å and a slippage of 1.92 Å.

## 4. Database survey

A search of the Cambridge Structure Database (CSD, version 6.00, last update April 2025; Groom *et al.*, 2016 ▸) revealed only one structure of the complex with a related anion (refcode UNURAA; Shum *et al.*, 2025 ▸), where a phenyl group is attached to the triazole ring instead of a benzyl group as in the title compound. The neutral 1-benzyl-5-thioxo-5,6-di­hydro-[1,2,4]triazolo[1,5-*c*]quinazolin-1-ium-2-thiol­ate was found as a solvate with DMF or DMSO in the crystal phase (Kovalenko *et al.*, 2026 ▸). A detailed comparison of the anion of (**I**) with related mol­ecules published previously is provided in the *Structural commentary*.

## 5. Synthesis and crystallization

The starting materials *N*-benzyl­thio­semicarbazide and 2-iso­thio­cyanato­benzo­nitrile are commercially available and, as well as the solvents, were purchased by Sigma Aldrich and used without further purification.

To a solution of 2-iso­thio­cyanato­benzo­nitrile (0.32 g, 2 mmol) in *i*-PrOH (15 ml), *N*-benzyl­thio­semicarbazide (0.32 g, 2 mmol) was added. Then a solution of NaOH (0.40 g, 10 mmol) in water (10 ml) was added and the reaction mixture was refluxed with stirring for 2 h. The next day, the grown yellow needle-like crystals were collected, washed with *i*-PrOH (5 ml) and dried at ambient temperature. Yield 0.53 g (66%). M.p. > 573 K. ^1^H NMR spectrum δ, ppm (*J*, Hz): 6.01 (2H, *s*, CH_2_); 7.11 (1H, *t*, *J* = 8.4, H Ar); 7.20–7.28 (3H, *m*, H-2,4,6 Ph); 7.31 (2H, *t*, *J* = 8.8, H-3,5 Ph); 7.45 (1H, *d*, *J* = 8.4, H Ar); 7.65 (1H, *t*, *J* = 8.4, H Ar); 7.81 (1H, *d*, *J* = 8.4, H Ar). ^13^C NMR spectrum, δ, ppm: 48.5 (CH_2_); 107.3; 121.6; 123.4; 124.4; 126.6 (2C); 128.0; 129.0 (2C); 134.2; 135.1; 142.4; 143.0; 164.5 (C-2); 172.0 (C-5). LC/MS *m*/*z* (*I*_rel_, %): 325.0 [*M* + H]^+^ (100). IR spectrum (KBr), ν, cm^−1^: 3508 (NH), 3335 (NH), 1614 (C=N), 1568 (C=N), 1367 (C=S polarized), 1173 (C=S). UV/Vis spectrum (MeOH), λ_max_ nm (ɛ): 255 (48000), 306 (59000), 364 (9800). Found, %: C 48.14; H 4.27; N 14.02; S 15.96. C_16_H_11_N_4_NaS_2_^.^3 H_2_O. Calculated, %: C 47.99; H 4.28; N 13.99; S 16.01.

The title compound was recrystallized by slow evaporation of a solution in aqueous DMF to produce colourless crystals suitable for X-ray diffraction analysis.

## 6. Refinement

Crystal data, data collection and structure refinement details are summarized in Table 3 ▸. Positions of hydrogen atoms bound to C atoms were calculated and refined as riding with *U*_iso_(H) = 1.2*U*_eq_(C). Positions of hydrogen atoms of water mol­ecules were discernible from difference-Fourier maps and refined with distance restraints of 0.90 (1) Å and with *U*_iso_(H) = 1.5*U*_eq_(O).

## Supplementary Material

Crystal structure: contains datablock(s) I. DOI: 10.1107/S2056989026005487/wm5796sup1.cif

Structure factors: contains datablock(s) I. DOI: 10.1107/S2056989026005487/wm5796Isup3.hkl

CCDC reference: 2110028

Additional supporting information:  crystallographic information; 3D view; checkCIF report

## Figures and Tables

**Table 1:** Selected bond lengths (in Å) in neutral compound (**I**) as solvates with DMF and DMSO (Kovalenko *et al.*, 2026 ▸), its deprotonated form in a complex with iridium (Shum *et al.*, 2025 ▸) and in the present structure

Bond	neutral (**I**), DMF	neutral (**I**), DMSO	anion (**I**) (complex with iridium)	anion (**I**) (this work)
N2—C8	1.342 (5)	1.334 (4)	1.308 (7)	1.312 (4)
C8—N3	1.395 (5)	1.399 (4)	1.415 (7)	1.411 (3)
N3—N4	1.374 (5)	1.386 (3)	1.387 (6)	1.382 (3)
N4—C9	1.319 (5)	1.317 (3)	1.337 (7)	1.318 (3)
C9—N1	1.421 (5)	1.417 (3)	1.422 (7)	1.399 (3)
N1—C1	1.353 (5)	1.340 (3)	1.334 (8)	1.355 (3)
C1—N3	1.349 (4)	1.349 (3)	1.333 (6)	1.342 (3)
C8—S1	1.644 (4)	1.651 (3)	1.712 (6)	1.687 (3)
C9—S2	1.685 (4)	1.683 (3)	1.659 (6)	1.697 (3)

**Table 2:** Hydrogen-bond geometry (Å, °)

*D*—H⋯*A*	*D*—H	H⋯*A*	*D*⋯*A*	*D*—H⋯*A*
O1—H1*A*⋯S1^i^	0.90 (1)	2.46 (2)	3.318 (2)	161 (3)
O1—H1*B*⋯N2	0.89 (1)	2.04 (1)	2.913 (3)	171 (3)
O2—H2*A*⋯S2^i^	0.90 (1)	2.38 (1)	3.261 (2)	168 (3)
O2—H2*B*⋯S1^ii^	0.89 (1)	2.37 (1)	3.253 (2)	170 (3)
O3—H3*B*⋯S1^iii^	0.90 (1)	2.96 (4)	3.599 (3)	130 (4)
O3—H3*B*⋯N4^iii^	0.90 (1)	2.09 (2)	2.947 (3)	159 (4)
C10—H10*A*⋯S2	0.97	2.72	3.200 (3)	111
C10—H10*B*⋯S2^iv^	0.97	2.77	3.684 (3)	158

Symmetry codes: (i) 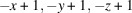; (ii) 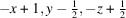; (iii) 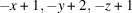; (iv) 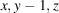.

**Table 3:** Experimental details

Crystal data	
Chemical formula	[Na(H_2_O)_3_](C_16_H_11_N_4_S_2_)
*M*_r_	400.44
Crystal system, space group	Monoclinic, *P*2_1_/*c*
Temperature (K)	296
*a*, *b*, *c* (Å)	17.4735 (10), 6.0363 (5), 17.106 (1)
β (°)	90.261 (5)
*V* (Å^3^)	1804.2 (2)
*Z*	4
Radiation type	Mo *K*α
μ (mm^−1^)	0.34
Crystal size (mm)	0.26 × 0.23 × 0.16

Data collection	
Diffractometer	Xcalibur, Sapphire3 CCD
Absorption correction	Multi-scan (*CrysAlis PRO*; Rigaku OD, 2024 ▸)
*T*_min_, *T*_max_	0.807, 0.881
No. of measured, independent and observed [*I* > 2σ(*I*)] reflections	13185, 3552, 2641
*R*_int_	0.081
(sin θ/λ)_max_ (Å^−1^)	0.617

Refinement	
*R*[*F*^2^ > 2σ(*F*^2^)], *wR*(*F*^2^), *S*	0.051, 0.145, 1.06
No. of reflections	3552
No. of parameters	253
No. of restraints	7
H-atom treatment	H atoms treated by a mixture of independent and constrained refinement
Δρ_max_, Δρ_min_ (e Å^−3^)	0.36, −0.28

Computer programs: *CrysAlis PRO* (Rigaku OD, 2024 ▸), *SHELXT* (Sheldrick, 2015*a* ▸), *SHELXL* (Sheldrick, 2015*b* ▸), *OLEX2* (Dolomanov *et al.*, 2009 ▸), *Mercury* (Macrae *et al.*, 2020 ▸) amd *publCIF* (Westrip, 2010 ▸).

## supplementary crystallographic information

### catena-Poly[1-benzyl-[1,2,4]triazolo[1,5-c]quinazolin-1-ium-2,5-bis(thiolate) [[aquasodium]-di-µ-aqua]] Crystal data

**Table:**

[Na(H_2_O)_3_](C_16_H_11_N_4_S_2_)	*F*(000) = 832
*M**_r_* = 400.44	*D*_x_ = 1.474 Mg m^−^^3^
Monoclinic, *P*2_1_/*c*	Mo *K*α radiation, λ = 0.71073 Å
*a* = 17.4735 (10) Å	Cell parameters from 2026 reflections
*b* = 6.0363 (5) Å	θ = 3.5–31.1°
*c* = 17.106 (1) Å	µ = 0.34 mm^−^^1^
β = 90.261 (5)°	*T* = 296 K
*V* = 1804.2 (2) Å^3^	Block, colourless
*Z* = 4	0.26 × 0.23 × 0.16 mm

### catena-Poly[1-benzyl-[1,2,4]triazolo[1,5-c]quinazolin-1-ium-2,5-bis(thiolate) [[aquasodium]-di-µ-aqua]] Data collection

**Table:**

Xcalibur, Sapphire3 CCD diffractometer	3552 independent reflections
Radiation source: Enhance (Mo) X-ray Source	2641 reflections with *I* > 2σ(*I*)
Detector resolution: 16.1827 pixels mm^-1^	*R*_int_ = 0.081
ω–scans	θ_max_ = 26.0°, θ_min_ = 3.3°
Absorption correction: multi-scan (CrysAlisPro; Rigaku OD, 2024)	*h* = −21→21
*T*_min_ = 0.807, *T*_max_ = 0.881	*k* = −7→7
13185 measured reflections	*l* = −21→21

### catena-Poly[1-benzyl-[1,2,4]triazolo[1,5-c]quinazolin-1-ium-2,5-bis(thiolate) [[aquasodium]-di-µ-aqua]] Refinement

**Table:**

Refinement on *F*^2^	Primary atom site location: dual
Least-squares matrix: full	Hydrogen site location: mixed
*R*[*F*^2^ > 2σ(*F*^2^)] = 0.051	H atoms treated by a mixture of independent and constrained refinement
*wR*(*F*^2^) = 0.145	*w* = 1/[σ^2^(*F*_o_^2^) + (0.0584*P*)^2^ + 0.1832*P*] where *P* = (*F*_o_^2^ + 2*F*_c_^2^)/3
*S* = 1.06	(Δ/σ)_max_ < 0.001
3552 reflections	Δρ_max_ = 0.36 e Å^−^^3^
253 parameters	Δρ_min_ = −0.28 e Å^−^^3^
7 restraints	

### catena-Poly[1-benzyl-[1,2,4]triazolo[1,5-c]quinazolin-1-ium-2,5-bis(thiolate) [[aquasodium]-di-µ-aqua]] Special details

**Table:**

Geometry. All esds (except the esd in the dihedral angle between two l.s. planes) are estimated using the full covariance matrix. The cell esds are taken into account individually in the estimation of esds in distances, angles and torsion angles; correlations between esds in cell parameters are only used when they are defined by crystal symmetry. An approximate (isotropic) treatment of cell esds is used for estimating esds involving l.s. planes.

### catena-Poly[1-benzyl-[1,2,4]triazolo[1,5-c]quinazolin-1-ium-2,5-bis(thiolate) [[aquasodium]-di-µ-aqua]] Fractional atomic coordinates and isotropic or equivalent isotropic displacement parameters (Å^2^)

**Table:**

	*x*	*y*	*z*	*U*_iso_*/*U*_eq_	
Na1	0.52548 (7)	0.57710 (18)	0.26179 (7)	0.0510 (4)	
S1	0.43289 (4)	0.81112 (13)	0.48604 (4)	0.0423 (2)	
S2	0.24556 (4)	0.91261 (12)	0.74807 (4)	0.0407 (2)	
O1	0.47490 (12)	0.3103 (3)	0.34950 (12)	0.0432 (5)	
H1A	0.5023 (17)	0.247 (5)	0.3877 (14)	0.065*	
H1B	0.4360 (14)	0.369 (5)	0.3755 (18)	0.065*	
O2	0.58758 (12)	0.2869 (3)	0.20286 (12)	0.0442 (5)	
H2A	0.6315 (11)	0.215 (5)	0.212 (2)	0.066*	
H2B	0.583 (2)	0.275 (6)	0.1510 (6)	0.066*	
O3	0.58277 (19)	0.8053 (5)	0.3537 (2)	0.1033 (12)	
H3A	0.610 (3)	0.785 (8)	0.310 (2)	0.155*	
H3B	0.607 (3)	0.922 (7)	0.375 (3)	0.155*	
N1	0.22627 (12)	0.5677 (3)	0.64514 (12)	0.0314 (5)	
N2	0.34848 (13)	0.4592 (4)	0.44675 (13)	0.0365 (5)	
N3	0.31405 (12)	0.6392 (4)	0.56116 (12)	0.0323 (5)	
N4	0.31986 (13)	0.8048 (3)	0.61663 (13)	0.0336 (5)	
C1	0.25761 (14)	0.4960 (4)	0.57742 (15)	0.0308 (6)	
C2	0.24173 (15)	0.3178 (4)	0.52590 (15)	0.0317 (6)	
C3	0.18493 (16)	0.1564 (5)	0.53539 (17)	0.0393 (7)	
H3	0.153124	0.161293	0.578796	0.047*	
C4	0.17595 (18)	−0.0082 (5)	0.48119 (17)	0.0442 (7)	
H4	0.138313	−0.115404	0.487870	0.053*	
C5	0.22324 (17)	−0.0149 (5)	0.41606 (17)	0.0425 (7)	
H5	0.216731	−0.126532	0.379162	0.051*	
C6	0.27924 (17)	0.1406 (5)	0.40553 (16)	0.0419 (7)	
H6	0.310374	0.132893	0.361671	0.050*	
C7	0.29033 (15)	0.3116 (4)	0.45996 (15)	0.0334 (6)	
C8	0.36250 (15)	0.6218 (5)	0.49565 (15)	0.0340 (6)	
C9	0.26555 (15)	0.7597 (4)	0.66746 (15)	0.0325 (6)	
C10	0.16773 (15)	0.4583 (5)	0.69313 (15)	0.0351 (6)	
H10A	0.171019	0.516422	0.745912	0.042*	
H10B	0.179185	0.301254	0.695582	0.042*	
C11	0.08730 (16)	0.4866 (5)	0.66410 (15)	0.0359 (6)	
C12	0.06367 (18)	0.6774 (5)	0.6272 (2)	0.0534 (9)	
H12	0.098599	0.790850	0.618382	0.064*	
C13	−0.0116 (2)	0.7022 (7)	0.6032 (2)	0.0694 (11)	
H13	−0.026672	0.831208	0.577738	0.083*	
C14	−0.0637 (2)	0.5386 (7)	0.6167 (2)	0.0700 (11)	
H14	−0.114160	0.554992	0.600091	0.084*	
C15	−0.0415 (2)	0.3522 (7)	0.6546 (2)	0.0642 (10)	
H15	−0.077268	0.241789	0.664687	0.077*	
C16	0.03406 (18)	0.3237 (5)	0.67847 (19)	0.0513 (8)	
H16	0.048621	0.194560	0.704180	0.062*	

### catena-Poly[1-benzyl-[1,2,4]triazolo[1,5-c]quinazolin-1-ium-2,5-bis(thiolate) [[aquasodium]-di-µ-aqua]] Atomic displacement parameters (Å^2^)

**Table:**

	*U*^11^	*U*^22^	*U*^33^	*U*^12^	*U*^13^	*U*^23^
Na1	0.0614 (9)	0.0388 (7)	0.0530 (8)	0.0054 (5)	0.0164 (6)	0.0040 (6)
S1	0.0385 (5)	0.0536 (5)	0.0348 (4)	−0.0138 (3)	0.0039 (3)	0.0001 (3)
S2	0.0459 (5)	0.0419 (4)	0.0344 (4)	0.0036 (3)	0.0017 (3)	−0.0062 (3)
O1	0.0457 (13)	0.0481 (13)	0.0358 (11)	0.0029 (10)	0.0050 (9)	0.0027 (9)
O2	0.0415 (13)	0.0561 (13)	0.0351 (11)	0.0075 (10)	0.0010 (9)	−0.0007 (10)
O3	0.087 (2)	0.097 (2)	0.126 (3)	−0.0412 (19)	0.023 (2)	−0.054 (2)
N1	0.0283 (12)	0.0352 (12)	0.0308 (11)	−0.0027 (9)	0.0015 (9)	−0.0011 (9)
N2	0.0320 (13)	0.0448 (13)	0.0328 (12)	−0.0031 (10)	0.0024 (10)	−0.0025 (11)
N3	0.0298 (12)	0.0363 (12)	0.0308 (12)	−0.0021 (9)	−0.0004 (9)	−0.0031 (10)
N4	0.0340 (13)	0.0328 (12)	0.0340 (12)	−0.0034 (9)	−0.0009 (10)	−0.0047 (10)
C1	0.0260 (14)	0.0355 (13)	0.0310 (14)	0.0004 (11)	−0.0011 (11)	0.0004 (11)
C2	0.0334 (15)	0.0321 (14)	0.0295 (14)	−0.0001 (11)	−0.0025 (11)	−0.0004 (11)
C3	0.0366 (16)	0.0424 (16)	0.0388 (15)	−0.0042 (12)	0.0039 (12)	−0.0029 (13)
C4	0.0467 (18)	0.0393 (15)	0.0466 (17)	−0.0080 (13)	−0.0006 (14)	−0.0029 (14)
C5	0.0471 (18)	0.0414 (16)	0.0390 (16)	−0.0014 (13)	−0.0051 (13)	−0.0117 (13)
C6	0.0463 (18)	0.0460 (16)	0.0336 (15)	−0.0008 (14)	0.0043 (13)	−0.0101 (13)
C7	0.0298 (15)	0.0390 (15)	0.0313 (14)	0.0022 (11)	−0.0023 (11)	−0.0004 (12)
C8	0.0319 (15)	0.0421 (15)	0.0280 (14)	−0.0001 (12)	0.0007 (11)	0.0001 (12)
C9	0.0306 (14)	0.0354 (14)	0.0314 (14)	0.0004 (11)	−0.0045 (11)	0.0006 (12)
C10	0.0350 (15)	0.0375 (14)	0.0328 (14)	−0.0012 (12)	0.0039 (12)	0.0027 (12)
C11	0.0342 (16)	0.0414 (15)	0.0321 (14)	−0.0032 (12)	0.0057 (11)	−0.0009 (12)
C12	0.0413 (19)	0.054 (2)	0.065 (2)	−0.0046 (15)	0.0017 (16)	0.0203 (16)
C13	0.046 (2)	0.078 (3)	0.084 (3)	0.0034 (19)	−0.0098 (19)	0.024 (2)
C14	0.040 (2)	0.097 (3)	0.073 (3)	0.002 (2)	−0.0089 (18)	0.003 (2)
C15	0.0387 (19)	0.079 (3)	0.075 (3)	−0.0187 (18)	0.0027 (17)	0.002 (2)
C16	0.0449 (19)	0.0522 (19)	0.057 (2)	−0.0078 (15)	0.0032 (15)	0.0109 (15)

### catena-Poly[1-benzyl-[1,2,4]triazolo[1,5-c]quinazolin-1-ium-2,5-bis(thiolate) [[aquasodium]-di-µ-aqua]] Geometric parameters (Å, º)

**Table:**

Na1—Na1^i^	3.1720 (9)	C1—C2	1.417 (4)
Na1—Na1^ii^	3.1720 (9)	C2—C3	1.400 (4)
Na1—O1^i^	2.368 (2)	C2—C7	1.416 (3)
Na1—O1	2.374 (2)	C3—H3	0.9300
Na1—O2^i^	2.426 (2)	C3—C4	1.368 (4)
Na1—O2	2.296 (2)	C4—H4	0.9300
Na1—O3	2.315 (3)	C4—C5	1.391 (4)
Na1—H3A	2.11 (5)	C5—H5	0.9300
S1—C8	1.687 (3)	C5—C6	1.368 (4)
S2—C9	1.697 (3)	C6—H6	0.9300
O1—H1A	0.896 (10)	C6—C7	1.403 (4)
O1—H1B	0.887 (10)	C10—H10A	0.9700
O2—H2A	0.895 (10)	C10—H10B	0.9700
O2—H2B	0.894 (10)	C10—C11	1.498 (4)
O3—H3A	0.900 (10)	C11—C12	1.375 (4)
O3—H3B	0.901 (10)	C11—C16	1.377 (4)
N1—C1	1.355 (3)	C12—H12	0.9300
N1—C9	1.399 (3)	C12—C13	1.384 (5)
N1—C10	1.471 (3)	C13—H13	0.9300
N2—C7	1.371 (3)	C13—C14	1.365 (5)
N2—C8	1.312 (3)	C14—H14	0.9300
N3—N4	1.382 (3)	C14—C15	1.354 (5)
N3—C1	1.341 (3)	C15—H15	0.9300
N3—C8	1.411 (3)	C15—C16	1.392 (5)
N4—C9	1.318 (3)	C16—H16	0.9300
			
Na1^ii^—Na1—Na1^i^	144.16 (9)	N1—C1—C2	134.1 (2)
Na1^ii^—Na1—H3A	144.3 (16)	N3—C1—N1	105.8 (2)
Na1^i^—Na1—H3A	71.4 (16)	N3—C1—C2	120.1 (2)
O1—Na1—Na1^i^	128.36 (8)	C3—C2—C1	126.4 (2)
O1^i^—Na1—Na1^ii^	117.63 (9)	C3—C2—C7	120.1 (2)
O1—Na1—Na1^ii^	47.93 (6)	C7—C2—C1	113.5 (2)
O1^i^—Na1—Na1^i^	48.11 (6)	C2—C3—H3	119.8
O1^i^—Na1—O1	155.74 (7)	C4—C3—C2	120.4 (3)
O1—Na1—O2^i^	83.72 (8)	C4—C3—H3	119.8
O1^i^—Na1—O2^i^	83.72 (8)	C3—C4—H4	120.1
O1^i^—Na1—H3A	87.8 (13)	C3—C4—C5	119.8 (3)
O1—Na1—H3A	114.8 (12)	C5—C4—H4	120.1
O2^i^—Na1—Na1^ii^	107.46 (8)	C4—C5—H5	119.6
O2^i^—Na1—Na1^i^	46.08 (6)	C6—C5—C4	120.9 (3)
O2—Na1—Na1^ii^	49.55 (6)	C6—C5—H5	119.6
O2—Na1—Na1^i^	143.42 (8)	C5—C6—H6	119.5
O2—Na1—O1^i^	95.75 (8)	C5—C6—C7	120.9 (3)
O2—Na1—O1	86.45 (8)	C7—C6—H6	119.5
O2—Na1—O2^i^	153.49 (8)	N2—C7—C2	124.2 (2)
O2—Na1—O3	123.27 (12)	N2—C7—C6	118.0 (2)
O2—Na1—H3A	107.0 (11)	C6—C7—C2	117.8 (2)
O2^i^—Na1—H3A	99.5 (11)	N2—C8—S1	125.34 (19)
O3—Na1—Na1^ii^	140.62 (12)	N2—C8—N3	116.8 (2)
O3—Na1—Na1^i^	68.92 (11)	N3—C8—S1	117.9 (2)
O3—Na1—O1^i^	101.07 (12)	N1—C9—S2	124.73 (19)
O3—Na1—O1	97.78 (11)	N4—C9—S2	125.1 (2)
O3—Na1—O2^i^	82.53 (11)	N4—C9—N1	110.2 (2)
O3—Na1—H3A	22.9 (3)	N1—C10—H10A	108.6
Na1^ii^—O1—Na1	83.97 (6)	N1—C10—H10B	108.6
Na1^ii^—O1—H1A	109 (2)	N1—C10—C11	114.7 (2)
Na1—O1—H1A	124 (2)	H10A—C10—H10B	107.6
Na1^ii^—O1—H1B	130 (2)	C11—C10—H10A	108.6
Na1—O1—H1B	110 (2)	C11—C10—H10B	108.6
H1A—O1—H1B	102 (3)	C12—C11—C10	121.8 (3)
Na1—O2—Na1^ii^	84.37 (7)	C12—C11—C16	118.6 (3)
Na1^ii^—O2—H2A	114 (2)	C16—C11—C10	119.5 (3)
Na1—O2—H2A	135 (2)	C11—C12—H12	119.7
Na1—O2—H2B	117 (2)	C11—C12—C13	120.6 (3)
Na1^ii^—O2—H2B	97 (2)	C13—C12—H12	119.7
H2A—O2—H2B	102 (3)	C12—C13—H13	119.8
Na1—O3—H3A	65 (3)	C14—C13—C12	120.4 (3)
Na1—O3—H3B	161 (4)	C14—C13—H13	119.8
H3A—O3—H3B	101.0 (15)	C13—C14—H14	120.2
C1—N1—C9	107.3 (2)	C15—C14—C13	119.5 (4)
C1—N1—C10	128.2 (2)	C15—C14—H14	120.2
C9—N1—C10	124.1 (2)	C14—C15—H15	119.6
C8—N2—C7	121.2 (2)	C14—C15—C16	120.8 (3)
N4—N3—C8	123.8 (2)	C16—C15—H15	119.6
C1—N3—N4	112.1 (2)	C11—C16—C15	120.0 (3)
C1—N3—C8	124.1 (2)	C11—C16—H16	120.0
C9—N4—N3	104.6 (2)	C15—C16—H16	120.0
			
N1—C1—C2—C3	−1.4 (5)	C5—C6—C7—N2	178.7 (3)
N1—C1—C2—C7	178.9 (3)	C5—C6—C7—C2	−0.1 (4)
N1—C10—C11—C12	−33.6 (4)	C7—N2—C8—S1	179.3 (2)
N1—C10—C11—C16	150.3 (3)	C7—N2—C8—N3	−1.2 (4)
N3—N4—C9—S2	−179.2 (2)	C7—C2—C3—C4	0.0 (4)
N3—N4—C9—N1	0.5 (3)	C8—N2—C7—C2	−0.7 (4)
N3—C1—C2—C3	179.9 (3)	C8—N2—C7—C6	−179.4 (3)
N3—C1—C2—C7	0.3 (4)	C8—N3—N4—C9	−179.3 (2)
N4—N3—C1—N1	−0.6 (3)	C8—N3—C1—N1	178.7 (2)
N4—N3—C1—C2	178.4 (2)	C8—N3—C1—C2	−2.3 (4)
N4—N3—C8—S1	1.6 (4)	C9—N1—C1—N3	0.9 (3)
N4—N3—C8—N2	−178.0 (2)	C9—N1—C1—C2	−177.9 (3)
C1—N1—C9—S2	178.8 (2)	C9—N1—C10—C11	107.0 (3)
C1—N1—C9—N4	−0.9 (3)	C10—N1—C1—N3	−173.0 (2)
C1—N1—C10—C11	−80.1 (3)	C10—N1—C1—C2	8.2 (5)
C1—N3—N4—C9	0.0 (3)	C10—N1—C9—S2	−7.0 (4)
C1—N3—C8—S1	−177.6 (2)	C10—N1—C9—N4	173.3 (2)
C1—N3—C8—N2	2.8 (4)	C10—C11—C12—C13	−177.9 (3)
C1—C2—C3—C4	−179.6 (3)	C10—C11—C16—C15	177.4 (3)
C1—C2—C7—N2	1.2 (4)	C11—C12—C13—C14	0.9 (6)
C1—C2—C7—C6	179.9 (3)	C12—C11—C16—C15	1.2 (5)
C2—C3—C4—C5	−0.3 (5)	C12—C13—C14—C15	0.6 (6)
C3—C2—C7—N2	−178.5 (3)	C13—C14—C15—C16	−1.2 (6)
C3—C2—C7—C6	0.1 (4)	C14—C15—C16—C11	0.3 (6)
C3—C4—C5—C6	0.4 (5)	C16—C11—C12—C13	−1.8 (5)
C4—C5—C6—C7	−0.2 (5)		

Symmetry codes: (i) −*x*+1, *y*+1/2, −*z*+1/2; (ii) −*x*+1, *y*−1/2, −*z*+1/2.

### catena-Poly[1-benzyl-[1,2,4]triazolo[1,5-c]quinazolin-1-ium-2,5-bis(thiolate) [[aquasodium]-di-µ-aqua]] Hydrogen-bond geometry (Å, º)

**Table:**

*D*—H···*A*	*D*—H	H···*A*	*D*···*A*	*D*—H···*A*
O1—H1*A*···S1^iii^	0.90 (1)	2.46 (2)	3.318 (2)	161 (3)
O1—H1*B*···N2	0.89 (1)	2.04 (1)	2.913 (3)	171 (3)
O2—H2*A*···S2^iii^	0.90 (1)	2.38 (1)	3.261 (2)	168 (3)
O2—H2*B*···S1^ii^	0.89 (1)	2.37 (1)	3.253 (2)	170 (3)
O3—H3*B*···S1^iv^	0.90 (1)	2.96 (4)	3.599 (3)	130 (4)
O3—H3*B*···N4^iv^	0.90 (1)	2.09 (2)	2.947 (3)	159 (4)
C10—H10*A*···S2	0.97	2.72	3.200 (3)	111
C10—H10*B*···S2^v^	0.97	2.77	3.684 (3)	158

Symmetry codes: (ii) −*x*+1, *y*−1/2, −*z*+1/2; (iii) −*x*+1, −*y*+1, −*z*+1; (iv) −*x*+1, −*y*+2, −*z*+1; (v) *x*, *y*−1, *z*.
